# Tissue specific transcript profiling of wheat phosphate transporter genes and its association with phosphate allocation in grains

**DOI:** 10.1038/srep39293

**Published:** 2016-12-20

**Authors:** Vishnu Shukla, Mandeep Kaur, Sipla Aggarwal, Kaushal Kumar Bhati, Jaspreet Kaur, Shrikant Mantri, Ajay K. Pandey

**Affiliations:** 1 National Agri-Food Biotechnology Institute (Department of Biotechnology, Government of India), C-127, Industrial Area, S.A.S. Nagar, Phase 8, Mohali-160071, Punjab, India; 2 Department of Biotechnology, University Institute of Engineering and Technology (UIET), Panjab University, Chandigarh, India

## Abstract

Approaches enabling efficient phosphorus utilization in crops are of great importance. In cereal crop like wheat, utilization of inorganic phosphate (Pi) is high and mature grains are the major sink for Pi utilization and storage. Research that addresses the importance of the Pi homeostasis in developing grains is limited. In an attempt to understand the Pi homeostasis in developing wheat grains, we identified twelve new phosphate transporters (PHT), these are phyologentically well distributed along with the members reported from Arabidopsis and rice. Enhanced expression of *PHT1*-subfamily genes was observed in roots subjected to the Pi starvation suggesting their active role in Pi homeostasis. Differential expression patterns of all the PHT genes during grain filling stages suggested their importance in the filial tissues. Additionally, high accumulation of Pi and total P in aleurone correlates well with the expression of *TaPHT*s and other phosphate starvation related genes. Tissue specific transcript accumulation of *TaPHT1.1, TaPHT1.2, TaPHT1.4* in aleurone; *TaPHT3.1* in embryo and *TaPHT4.2* in the endosperm was observed. Furthermore, their transcript abundance was affected in low phytate wheat grains. Altogether, this study helps in expanding the knowledge and prioritize the candidate wheat Pi-transporters to modulate the Pi homeostasis in cereal grains.

Phosphorus (P) is an essential mineral nutrient for plant growth and development^1^. Seeds of cereal and legume crops are major “sink” for P over-accumulation which consequently results in over-consumption of total P fertilizer used worldwide^2^. Due to low phytoavailability of inorganic or free phosphate (Pi), in a sustainable agricultural production, it is important to consider the contribution offered by the stored seed P. In general Pi acquisition occurs through the roots of the plants and subsequently transported to the stem, leaves and other tissue. Remobilization of Pi takes place in the foliar parts of the plants and subsequently majority of it gets stored into the developing grains as phytic acid (PA)^3^. Therefore, any variation in Pi acquisition, distribution and redistribution during plant development will significantly alter the seed total P during cereal grain development^4^. Due to the absence of symplasmic linkage between maternal and filial generations, once remobilized Pi reaches seed apoplasm, subsequent Pi transport totally depends on filial tissue functions which may impact net seed total P^5^,^6^.

Pi transport from the rhizosphere to the different plant tissues, primarily involves multiple phosphate transporters (PHTs) belonging to either PHT1, PHT2, PHT3 or PHT4 sub-families^7^,^8^,^9^,^10^,^11^. Most of the studies till date addressed the efficient utilization of soil P and its uptake by the roots via these transporters^12^,^13^,^14^. The regulation of PHT1 candidates involves the Pi starvation response regulators (PHR1) through their binding to *cis*-element P1BS (PHR1 binding sequence), SPX-proteins and PHO2 regulation^15^,^16^,^17^. Studies in rice (*Oryzae sativa*) and *Arabidopsis (Arabidopsis thaliana*) have suggested the role of *PHT1* family genes in remobilization of P from senescing tissues to the actively developing tissues and their role in embryo development^18^,^19^. In addition to that, Pi transport within the plant tissues is also contributed by PHT3 and PHT4 family members by regulating ‘intracellular Pi starvation’ signalling^20^,^21^. Earlier, it has been proposed that the P translocation and subsequent loading in grains is a demand-driven process^22^. However, researchers have to yet explore the mechanism involved for sensing the Pi loading in seed tissue during grain-filling.

In cereals during the early stages of maturation, Pi is transported in the seeds and rapidly converted to the bound form, commonly referred as PA^2^,^23^. The acidic nature of PA enables chelation of important micronutrients in form of reservoir generally referred as phytin bodies^24^,^25^. In grains, Pi is transported through pericarp, containing vascular compartment embedded in ground tissues. Subsequently, it is delivered to the tissues surrounding the developing seed such as, nucellar projection cells in wheat (*Triticum aestivum*) and barley (*Hordeum vulgare*)^4^. Once Pi gets deposited into the endosperm cavity (especially in case of wheat), the uptake process at the maternal/filial interface is adapted by specialized filial cells, such as “endosperm/aleurone transfer cells” or the cells of the embryo-surrounding region^5^.

Wheat is an agronomical important crop and a major source of nutrition in the developing countries, but it utilizes large amount of exogenous P during cultivation. P in addition to its role in the agronomic quality of grain also promotes uniform heading and faster maturity^26^. The acquired soil P, is vital for plant development that supports the germination of wheat seedling and vegetative growth till maturity. The high accumulation of PA in grains also suggests the presence of a controlled regulatory mechanism for Pi-PA homeostasis that could be active during seed development stages^27^,^28^. In order to develop Pi-efficient wheat, it is therefore important to understand the process that involves the Pi homeostasis and allocation in the developing grains of wheat. Despite the presence of clues related to the role of seed Pi, limited efforts were undertaken to address the same^22^,^29^. Therefore, it is important to understand the step-wise regulatory mechanism for the P accumulation in the wheat seed tissues. In this study, we identified 23 wheat PHT including 12 new members that span all the four sub-families of transporters and were further characterized for their expression in filial tissues during the grain filling stages. Expression of wheat *PHT*s was also studied in low phytate wheat, that suggested Pi-PA homeostasis in developing grains.

## Results

### Pi concentration in developing wheat seeds and tissues

**Figure 1:**
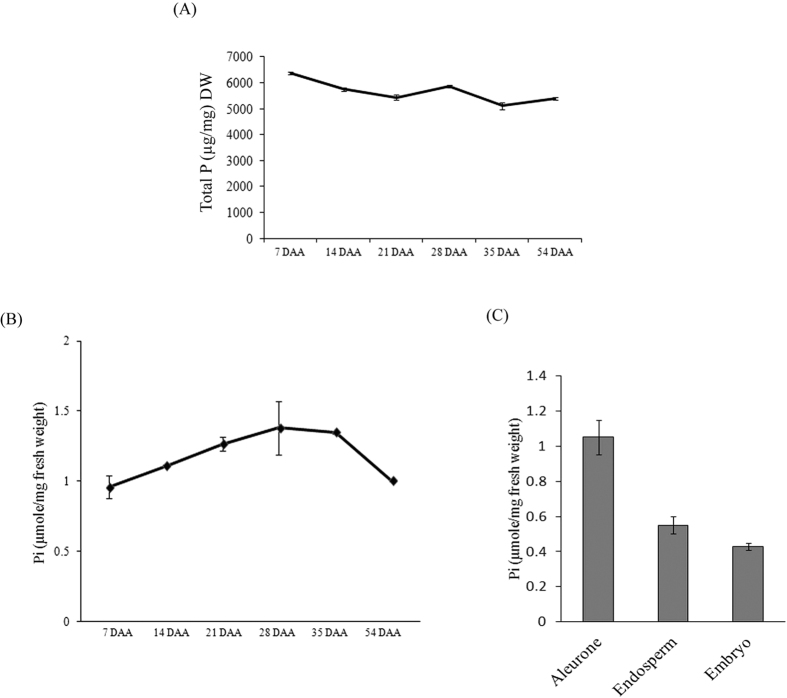
Estimation of inorganic phosphate (Pi) and total P in wheat seeds. Measurement of Pi and total phosphorous in wheat seeds. (**A**) Total P content in developing seeds. (**B**) Pi content in developing wheat seeds mainly, 7, 14, 21, 28, 35 DAA and mature seed (MS) (**C**) Pi content in 14 DAA aleurone (Al), endopserm (En) and embryo (Em). Each bar represents the mean ± SE of at least three biological replicates.

To compare the Pi accumulation in the developing grains, Pi and total P estimation was performed in the wheat grains. The concentration of total P throughout seed development remained stable with the exception for 7 DAA where enhanced accumulation was observed (Fig. 1A). Our analysis showed a slight increase in total Pi accumulation from 7 to 28 DAA of grain development (Fig. 1B). During this duration of grain filling, the concentration of Pi ranges from ~ 0.9–1.3 μmol/mg fresh weight. After 28 DAA, Pi level was slightly decreased at the maturation stage. This constant accumulation of Pi during the grain maturation reinforce that the developing grains are the sink tissue for phosphate storage. Next, Pi was analyzed in the filial tissues of 14 DAA wheat grains. Pi accumulation in aleurone tissue was 2-fold higher when compared to endosperm and embryo (Fig. 1C). The total P concentration was also 3-fold higher in aleurone in comparison with endosperm (Supplementary Fig. 1). This analysis suggests an existence of a Pi gradient in the filial tissues of developing wheat grains with over-accumulation of Pi in aleurone as compared to other tissue. These results also support our speculation for the possible role of PHTs those could be involved in the phosphate related homeostasis during grain development.

### *In-silico* identification and structural analysis of wheat PHTs

Previously, only eight wheat PHTs were reported^30^. To identify additional wheat PHTs, *Arabidopsis* (PHT1–19) and rice (PHT1-26) sequences were used as query to perform tBLASTN analysis. Subsequently, sequence alignment and unigene BLAST analysis was used to assemble 492 wheat ESTs into 23 different wheat genes (Supplementary Table 1). Their predicted intron-exon structure are represented in Supplementary Fig. 2. The nomenclature of identified 23 wheat PHTs was based on the sequence similarity to closest rice homologs. The analysis resulted in the identification of 12 additional wheat PHTs those cover all the subgroups of transporters.

**Figure 2:**
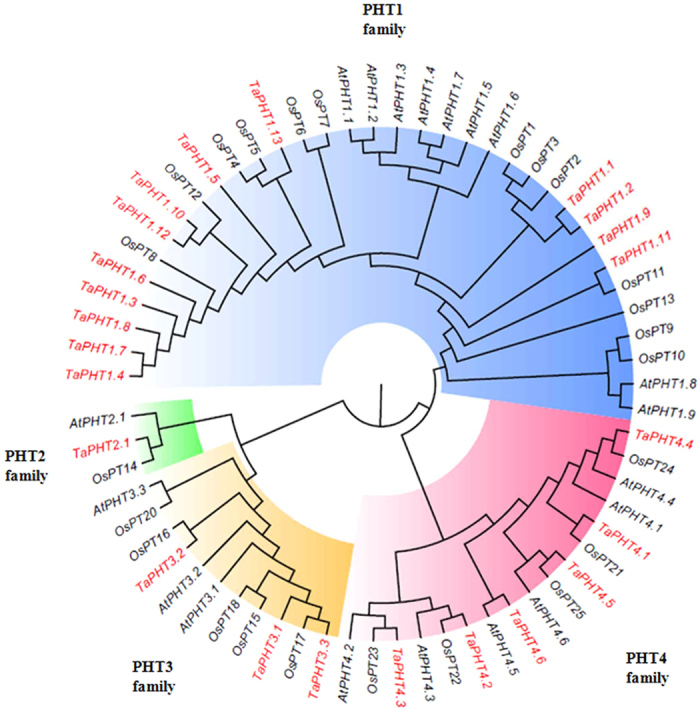
Phylogenetic relationship between the identified wheat PHT proteins along with PHT proteins from *Arabidopsis thaliana* and *Oryzae sativa.* Analysis was performed by Neighbour-joining tree analyses conducted using Mega5^58^. Bootstrap values were calculated as a percentage of 1000 trial. The analysis involved 68 protein sequences. The GeneBank accession numbers of the protein are listed in Supplementary Table 3.

**Figure 3:**
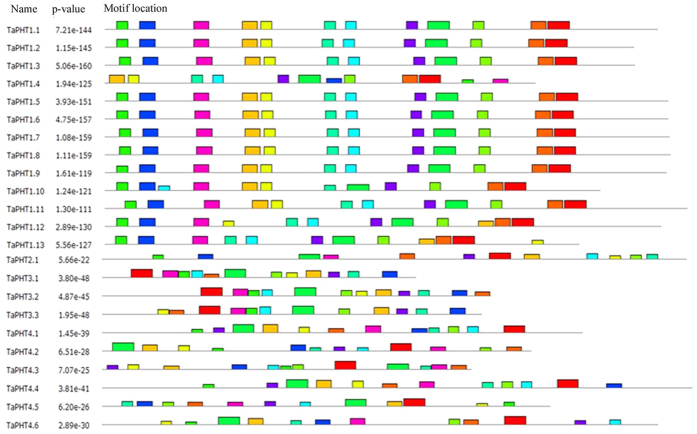
The protein architecture of *TaPHTs* displaying conserved motifs among wheat PHT family members. Analysis was performed in Multiple Em for Motif Elicitation (MEME) SUITE 4.10.2.

Phylogenetic tree developed from the protein sequence alignments of PHT family members distributed the wheat PHTs into four families (PHT1–PHT4) like in *Arabidopsis*^8^ and Rice^10^. Cluster I contains 13 wheat genes (*TaPHT1.1-1.13*) from PHT1 family; cluster II contains previously reported *TaPHT2.1* from PHT2 family; cluster III have 3 wheat genes (*TaPHT3.1-3.3*) of PHT3 family; and lastly cluster IV consisted of 6 wheat genes (*TaPHT4.1-4.6*) belonging to the PHT4 family (Fig. 2). Transport classification database (www.tcdb.org/analyze.php) revealed the presence of 4-12 transmembrane domains (TMs) on the predicted 23 TaPHT proteins (Supplementary Table 1 and Fig. 3). Similar to rice and *Arabidopsis*, almost all wheat PHT1 transporters having 12 TM domains clustered together (cluster I) except for *TaPHT1.10* (9 TMs) and *TaPHT1.12* (10 TMs; Supplementary Fig. 3). Distribution of *TaPHT2.1* in cluster II containing 13 TM domains was in agreement with the previous reports^9^ (Supplementary Fig. 4). Whereas, the members of *TaPHT3* transporters (cluster III) were found to have only 5 TM regions (Supplementary Fig. 5). Lastly, in cluster IV, TaPHT4 family members contains 12 TM domains, whereas, members from rice and *Arabidopsis* have 11–12 and 10–12 TM domains respectively (Supplementary Fig. 6). Our analysis suggests that the protein architecture of TaPHT sub-family members was conserved among the sub-family (Fig. 3).

### Genomic location and expression of wheat PHTs during Pi starvation

Chromosomal locations for all the TaPHT members were identified using IWGSC database (Supplementary Table 1). The wheat PHTs were distributed mainly on chromosome 4 (8 PHT genes), chromosome 5 (6 PHT genes), chromosomes 3 and 6 (3 PHT genes on each) and chromosomes 1 and 2 (2 PHT genes each) (Supplementary Table 1). In an attempt to get insight for the regulation of these wheat *PHT*s, the 1-kb upstream regions of all the wheat PHT genes were analyzed for the presence of *cis* elements. As expected, the searches revealed presence of multiple phosphate, hormone, sugar and stress responses related cis elements (Supplementary Table 2). Among these 82 putative *cis*-elements, 14 *cis*-elements grouped into Pi-related responsiveness, hormone-response, sugar-response and stress-response. Interestingly, 11 of 23 wheat PHT genes were found to contain the P1BS DNA motif (GNATATNC) on their promoter region (Supplementary Table 2). Among wheat PHT1 sub-family genes only six members have the phosphate responsive domain. The promoter of only three members of *TaPHT3* sub-family, *TaPHT2.1* and *TaPHT4.2* contains a P1BS binding motif.

**Figure 4:**
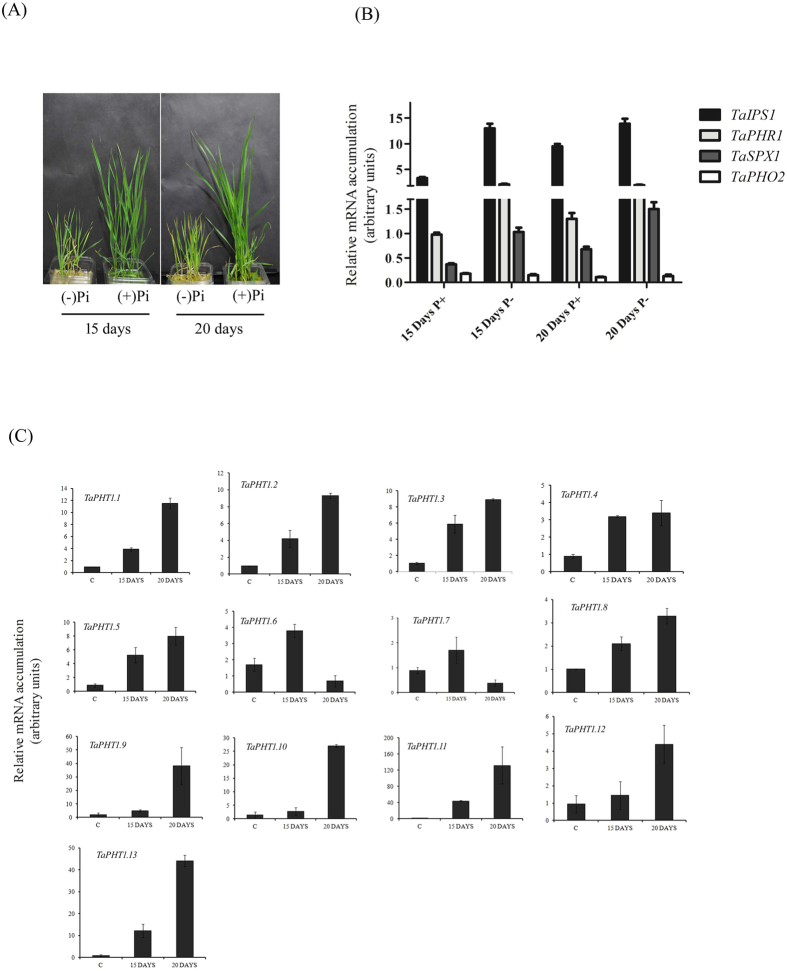
Quantitative real-time PCR expression analysis of putative wheat phosphate transporter genes and PSR marker genes under phosphate starvation. Expression was performed for PHT1 family (*TaPHT1.1-1.13*) and PSR marker genes (*TaIPS1, TaPHR1, TaSPX1* and *TaPHO2*) under 15 days and 20 days of Pi starvation. (**A**) Growth of wheat plant and concentration of Pi under Pi-starvation. (**B**) Transcript abundance of PSR marker genes. (**C**) Relative expression of TaPHT1 family members under Pi-limiting condition. Each bar indicates the mean of four to five replicates with the indicated standard deviation of the mean.

The expression pattern of wheat PHT1 subfamily was examined in roots at two different time points (15 and 20 days) of Pi starvation (Fig. 4A). qRT-PCR analysis were carried out using gene specific conserved primers targeting the conserved sequence of transcripts arising from multiple genomes (A,B or D). During our experiment no significant changes in the expression of wheat PHTs were observed in the control plants at 15 and 20 days; therefore, the fold change in expression of wheat PHTs was calculated *w.r.t.* 15 days control samples. Firstly, expression response of phosphate starvation response genes (PSR) was tested for the Pi starvation in roots (Fig. 4B). Results suggested high expression of PSR related genes in roots suggesting a starvation response from the plants. Next, the expression of all the PHT1 sub-family genes was studied (Fig. 4C). In roots, wheat PHT1 subfamily of genes showed a distinct expression pattern that could be divided into three types of responses. Most of the genes followed, type-I response that was characterized by an incessant increase in the gene transcript at both 15 and 20 days of starvation. This expression pattern was observed for eight wheat PHTs including *TaPHT1.1, TaPHT1.2, TaPHT1.3, TaPHT1.5, TaPHT1.8, TaPHT1.11, TaPHT1.12* and *TaPHT1.13*. Second,type-II expression was characterized by induction at specific time point. For example, *TaPHT1.6* and *TaPHT1.7* were induced at 15 days, whereas, *TaPHT1.9* and *TaPHT1.10* were induced at 20 days after starvation. Lastly, in the type-III response, induction level of was similar at both the time points as in case of *TaPHT1.4*. The wheat Pi-transporters were also differentially expressed in shoots (data not shown). Summarizing the expression analysis, it was observed that members of wheat PHT1 subfamily showed transcriptional changes under Pi starvation condition.

### Expression of wheat PHTs in developing grains and tissue

**Figure 5:**
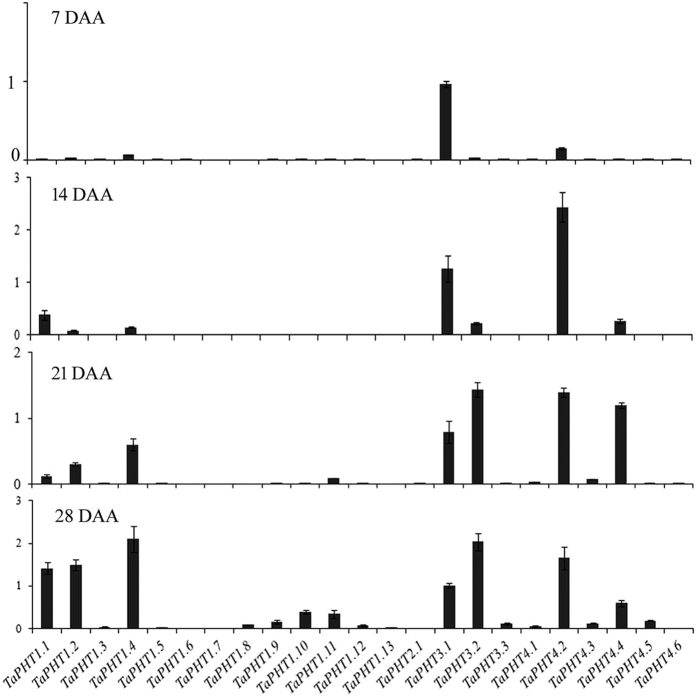
Relative transcript levels of 23 *TaPHTs* during seed development. The cDNA templates were prepared from 2 μg of DNA free RNA isolated from different time point of seed maturation of 7, 14, 21 and 28 DAA. qRT-PCR expression was performed for *TaPHT1.1-1.13, TaPHT2.1, TaPHT3.1-3.3* and *TaPHT4.1-4.6* during grain filling stages. Each bar indicates the mean of four to five replicates with the indicated standard deviation of the mean.

Expression of few Pi-homeostasis related genes, including PHTs was earlier reported in wheat seedlings subjected to limited Pi conditions^31^. Therefore, one could anticipate the presence of Pi and PA in developing grains that might influence the expression patterns of these genes. In the current study, we analyzed the expression at different stages of seed development (7, 14, 21 and 28 DAA). Our analysis suggested differential expression of these genes during grain maturation (Fig. 5). For example, at the early stage of development, i.e. 7 DAA, only *TaPHT3.1* and *TaPH4.2* showed significantly higher expression compared to other transporters. Similarly, at 14 DAA transcript abundance of *TaPHT3.1, TaPH4.2* and *TaPHT1.1* was higher compared to other transporters. At 21 DAA, multiple wheat PHTs have a high expression, including *TaPHT1.4, TaPHT3.1, TaPH3.2, TaPHT4.2* and *TaPHT4.4*. At the 28 DAA, *TaPHT1.1, TaPHT1.2, TaPHT1.4, TaPHT3.1, TaPHT3.2, TaPHT4.2* and *TaPHT4.4* were highly expressed. These data suggested active expression of wheat PHTs during grain development. In addition to that, at 28 DAA, maximum number of wheat PHTs were up-regulated. The differential expression patterns of wheat PHTs and PA pathway genes^32^, suggested interlink between Pi transport and PA biosynthesis.

**Figure 6:**
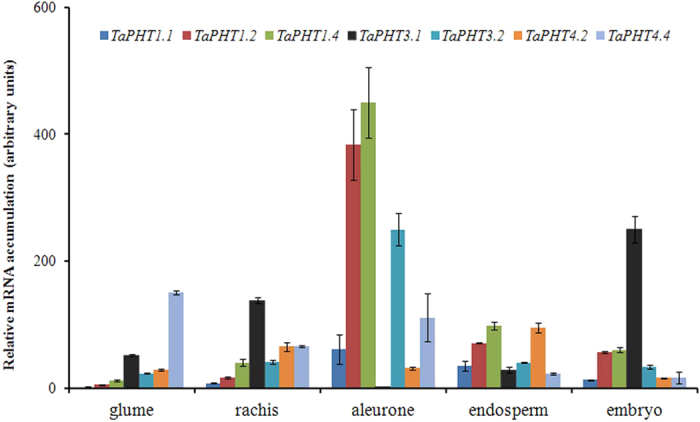
Transcript accumulation of candidate *TaPHTs* in 14 DAA spike and seed tissues. Relative expression of *TaPHT1.1, TaPHT1.2, TaPHT1.4, TaPHT3.1, TaPHT3.2, TaPHT4.2* and *TaPHT4.4* was analyzed in Glume (Glu), Rachis (Ra).Aleurone (Al), endosperm (En) and embryo (Em) of the 14 DAA old tissues. Each bar indicates the mean of four to five replicates with the indicated standard deviation of the mean.

To gather the evidence for the role of PHTs in the Pi-allocation within the seed compartments, highly expressed wheat PHTs (*TaPHT1.1, TaPHT1.4, TaPHT1.2, TaPHT3.1, TaPHT3.2, TaPHT4.2* and *TaPHT4.4)* were further selected. Tissue specific expression analysis of these genes was performed in glume, rachis, aleurone, endosperm and embryo of 14 DAA seed. Result suggested specific expression patterns of wheat PHTs (Fig. 6). For instance, expression of *TaPHT1.2* and *TaPHT1.3* was high in aleurone tissue, whereas, *TaPHT3.1* mRNA was significantly expressed in embryo and rachis. Unlike others, transcript abundance of *TaPHT4.2* and *TaPHT4.4* was highest in endosperm. The above expression analysis suggested the possibility of divergent function of PHTs in a tissue-specific manner.

### Expression response of PSR related genes in wheat filial tissue

**Figure 7:**
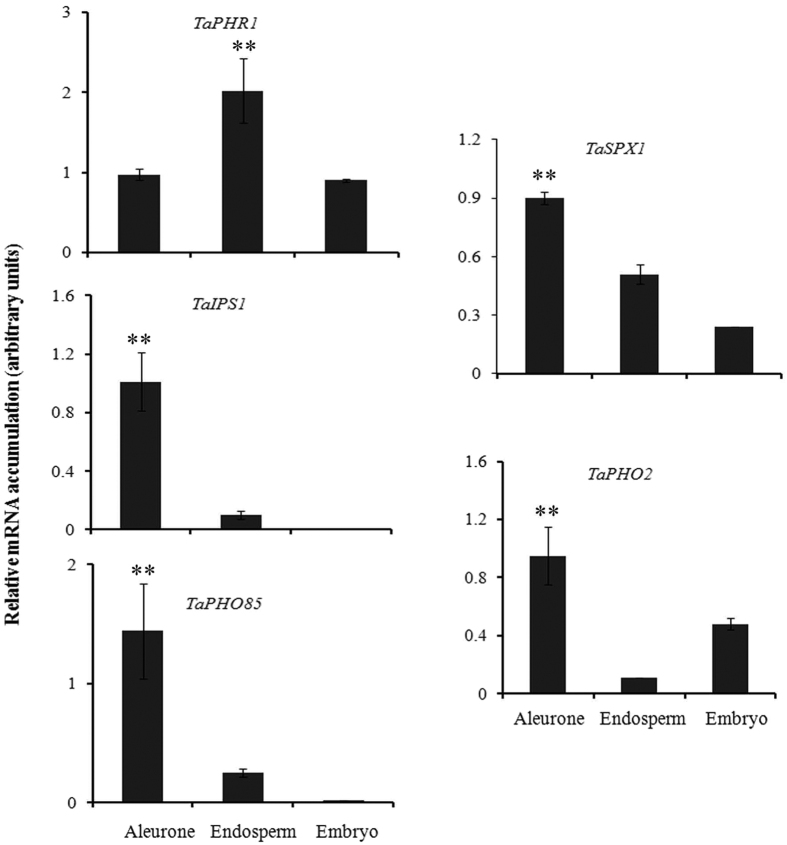
Influence of seed development on transcript levels of phosphate starvation response (PSR) related genes. The cDNA templates were prepared from 2 μg of DNA free RNA isolated from different tissues of 14 DAA wheat grain. Expression was studied for representative PSR related genes i.e. *TaPHR1, TaSPX1, TaIPS1* and*TaPHO2* at grain filling stage. Each bar indicates the mean of four to five replicates with the indicated standard deviation of the mean. **Indicates significant differences at p < 0.05 w.r.t. other tissue (Annova, Origin 6.1).

The tissue specific expression patterns and functionality of the wheat PHTs, suggested the coordinated response of these transporters. Our analysis also suggested high accumulation of Pi in aleurone tissue as compared to embryo and endosperm. Therefore, we speculate that the PSR related genes might also participate in controlling the accumulation of Pi in wheat tissue. Expression of the PSR related genes was tested in different tissues of wheat grains (Fig. 7). Expression characterization was performed for known PSR related genes, i.e., *TaPHR1, TaSPX1, TaPHO2, TaPHO85* and *TaIPS1*. qRT-PCR revealed that the relative transcript levels of *TaSPX1, TaPHO2, TaPHO85* and *TaIPS1* were significantly higher in aleurone in comparison to endosperm and embryo. Surprisingly, transcript level of *TaPHR1* was suppressed in the aleurone and embryo, but was significantly higher in endosperm of 14 DAA grains. Such contrasting, expression of PSR related genes in the wheat filial tissue suggested the importance of Pi-starvation signaling events and interplay of wheat *PHT*s in seed tissue.

### Lowering in phytic acid affects the expression of wheat PA biosynthesis genes and PHTs

**Figure 8:**
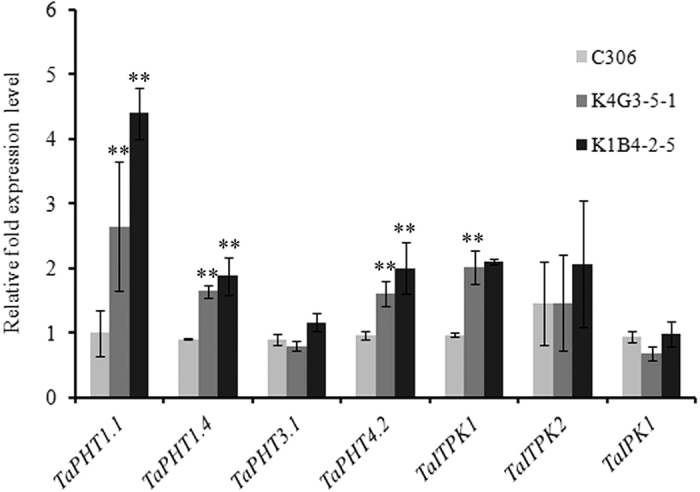
Relative expression levels of selected wheat Pi-transporters (*TaPHT1.1, TaPHT1.4, TaPHT3.1* and *TaPHT4.2*) and PA biosynthesis genes. The cDNA templates were prepared from 2 μg of DNA free RNA isolated from different tissues of 14 DAA wheat grain of C306 (non-transgenic) and two lines of transgenic wheat with low PA (K4G3-1-5-1 and K1B4-2-5). Each bar indicates the mean of three replicates with the indicated standard deviation of the mean. **Indicates significant differences at p < 0.05 w.r.t. to C306 (Annova, Origin 6.1).

In an attempt to gain insight for the Pi-PA homeostasis in wheat grains, we utilized the previously developed low phytate transgenic wheat^33^. The expression of the selected PA biosynthesis genes and Pi-transporters were performed in the 14 DAA seeds of two different low phytic acid lines (K4G3-5-1 and K1B4-2-5). Perturbation in the expression patterns of the selected genes was observed as compared to the non-transgenic seeds of C306. The transcripts of *TaPHT1.1, TaPHT1.4* and *TaPHT4.2* were largely affected (Fig. 8). Higher expression of these genes was observed in transgenic seeds. For the genes involved in PA biosynthesis, *TaIPK1* and *TaITPK2* did not show any significant difference in the expression level, whereas, *TaITPK1* showed high expression in both the transgenic plants. These experiments suggest that lowering of PA selectively affects the transcript level of genes involve in Pi transport and PA biosynthesis.

## Discussion

Wheat utilizes a substantially large amount of Pi, which is stored in seeds as PA. Cereal grains are an important reservoir of P that could be exploited for plant development. Not many reports related to the tissue specific compartmentalization of Pi in wheat seed tissues have been addressed. In order to study this, we report here first comprehensive analysis of wheat PHT transporter gene family with emphasis upon the tissue specificity and drawing clues regarding their possible function. Given the close proximity with the *Arabidopsis* and rice homologs, we expect that the wheat PHTs should also perform similar functions. Herein, we identified seed-specific Pi transporters, those could be the potential target to modulate the influx in developing cereal grains.

For wheat, the major source of total grain P is directly influenced by Pi transport from vegetative organs^34^. The translocation of Pi into the developing grains fulfills the requirement for cellular function and further storing as a reservoir in the form of phytate to be utilized for nutritional requirement during germination^35^. Previous studies in developing rice seed suggested that total P is concomitantly accumulated during 6 to 14 DAA and remained stable till maturation, whereas concentrations of Pi remains same throughout seed development with a slight decrease towards the maturation^36^. In our study, total P concentration was highest at 7 DAA and after a slight decrease it remained stable till maturation. This suggests a maximum utilization of incoming Pi during the early developmental stage. In general, the concentration of Pi during wheat seed development remained invariably constant. The Pi accumulation pattern during rice grain development was also shown to be constantly low^36^. Therefore, one can speculate that during wheat seed development, constant lower levels of Pi till 28 DAA is maintained by continuous translocation of Pi from vegetative tissues into developing seeds. However, the source tissue for providing available Pi to rice and wheat grains during development might differ due to difference in senescence rate of vegetative tissues^34^. Pi is a potent inhibitor of ADP-Gluc pyrophosphorylase, a key regulatory enzyme in starch biosynthesis^37^,^38^. Thus, during seed development high starch levels in wheat endosperm must be correlated with low concentrations of Pi.

For cereal grains, limited reports are available that describe the co-relation between Pi concentrations and its subsequent movement into the filial tissues^19^,^22^. In wheat, two principal filial tissues, aleurone and endosperm are involved in a biochemical and transcriptional reprogramming during grain filling to facilitate nutrient transport^39^,^40^.The total P concentration was found to be very high in aleurone as compared to endosperm of the 14 DAA seed, the stage when aleurone is discrete (Fig. 1C and Supplementary Fig. 1). Our analysis suggested that the aleurone accumulate more Pi than endosperm. The reason of differential Pi gradient in these tissues is still unknown, however, two inferences can be more acceptable on the basis of previous studies. Firstly, at 14 DAA, favorable translocation of more Pi in aleurone is required for actively accumulating phytic acid; secondly the unfavorable translocation of Pi in the endosperm is to promote starch biosynthesis to keep ADP-Gluc pyrophosphorylase active. Therefore, this comprehensive expression analysis of *TaPHTs* and Pi-homeostasis related genes in these tissues may provide better insight about Pi-transport and regulation mechanism during grain filling. During our study, we observed that aleurone accumulated more Pi with high expression of *TaPHT* genes (especially *PHT1* members) and PSR genes. Thus, basis of *TaPHT*s expression in aleurone is uncertain, as it accumulates more phosphorous than other tissue. Does Pi starvation responses govern the Pi transport in seed tissues similar to root and shoot? This remains an open question. High phytic acid content and expression of wheat PA pathway genes in aleurone^32^ are major factors for its physiological difference from roots and shoots w.r.t. Pi homeostasis. Overexpression of PA biosynthesis genes in rice resulted in an enhanced influx of P from vegetative organs into seeds^41^. These observations support the hypothesis for the continuous uptake of Pi that is translocated to aleurone layer, where it is rapidly utilized for the biosynthesis of PA.

Previous studies, have reported the identification of only eleven wheat *PHTs* (mainly, *TaPT1-8; TaPHT2.1*) and their expression profiles under Pi-starvation^9^,^30^,^42^. In this study, additional members of PHTs were included and their expression patterns were studied during seed development (mainly 7, 14, 21 and 28 DAA). To our knowledge, no homologs of PHT3 and PHT4 genes were previously reported from wheat. Recent reports provided the functional clues for multiple rice PHTs, suggesting that certain Pi-transporter (*OsPT1, OsPT8, OsPT14* and *OsPT18*) modulate the Pi transport within the seed tissues^22^. Based on these evidences and our results, the high expression response of *TaPHT1.1, TaPHT1.2, TaPHT1.4, TaPHT3.1, TaPHT3.2, TaPHT4.2* and *TaPHT4.4* suggests the importance of wheat transporters in the specific seed tissue (Fig. 6). Knockdown of *OsPT8,* a rice phosphate transporter; restricted the Pi allocation into embryo and affected the total P concentrations^43^. These observations also reinforce the possibility to target specific PHTs for gene silencing to modulate Pi balance in wheat. Analysis of aleurone and endosperm transcriptome data from previous reports^39^ showed several fold induction of PHT genes belonging primarily to PHT1 and PHT3 family. This supports our observations about the high abundance of Pi transporters in aleurone. Membrane-related transport activity has been found to be important in the wheat seed development specially near crease region and nucellar projections after phloem unloading^44^,^45^. *In-situ* PCR for *TaPHT1.1* transcript near chalazal region show importance of this tissue interface in transport of Pi within filial tissues (Supplementary Fig. 7). In our study, high transcript abundance of *TaPHT1.1, TaPHT1.2, TaPHT1.4* and *TaPHT3.2* was observed in aleurone (including crease region). Thus, it seems that the increased expression of multiple PHTs, might be as a consequence of enhanced accumulation of Pi in aleurone tissue. During grain development, accumulated Pi gets utilized for the synthesisof PA through a series of enzymes like inositol phosphate kinases in a lipid dependent or independent manner^3^,^32^. Therefore, maintaining the flux of Pi for phosphorylation series of *myo*-inositol moieties may be one of the probable roles of PHT members in the aleurone.

Endosperm being a storage tissue involved actively in starch synthesis^46^. Pi, PPi, ATP and ADP tightly regulate both starch synthesis and degradation in wheat endosperm cell^47^,^48^. In this study, we found the expression of *PHT4.2* and *PHT3.1* in wheat endosperm and embryo, respectively. Thus, we could hypothesize that the low Pi concentration regulated by PHTs plays important role in regulating starch biosynthesis in the endosperm. Although not conclusive, further experiments are required to confirm this hypothesis. Similarly, wheat PHT3 could have an involvement in the stress-related or other metabolic related functions in the embryo^49^.

*lpa* mutants have provided few clues for the Pi accumulation in grains. However, the link between inositol signalling pathway and Pi transport in seed tissues is still unknown^4^. Our results suggest that lowering of PA in wheat grains, affects the expression of PA biosynthesis and Pi-transporter related genes in wheat (Fig. 8). Previous studies on wheat has shown that lowering of PA eventually decreases the total P in the aleurone with increased total P in the inner endosperm^50^. In our case, the changes in the expression of wheat *PHTs* in *lpa* wheat grains could be as a result of varying P fractions forms in the grain tissues. These observations were supported from the recent studies that showed the involvement of genes related to Pi signalling; sulfur metabolism and sulphate transport; and in PA metabolism^51^. Therefore, the co-relation between Pi-PA homeostasis is of high importance in developing cereal grains.

The present study provides the comprehensive genomic analysis and spatio-temporal expression characterization of wheat PHTs and further explored their possible role in the filial tissue. The identified Pi-transporters could therefore be a potential candidate for gene targeting to modulate the gradient of Pi and biosynthesis of PA in seeds. Our data also contribute in laying the foundation towards understanding the molecular basis of Pi homeostasis in wheat developing grains. Overall, this study substantiates the role of Pi-transporters in uptake and regulating the optimal balance of Pi and thereby participates in controlling the Pi-PA homeostasis in wheat seed compartments.

## Materials and Methods

### Identification of PHT genes in wheat genome and sequence analysis

The *in-silico* identification of PHT genes from wheat was adapted from the approach reported previously^52^. The protein sequences of PHT family genes (PHT1, 2, 3 and 4) from *Arabidospsis* and rice (Supplementary Table 3) were queried against wheat expressed sequence tags (EST) database in tBLASTn program at NCBI (http://blast.ncbi.nlm.nih.gov/blast/Blast.cgi). All the different EST accessions were queried in Unigene (http://www.ncbi.nlm.nih.gov/unigene) for assigning unique Unigene ID to particular set of ESTs. These ESTs were mapped individually on cereal data base (DB) (http://www.cerealsdb.uk.net/cerealgenomics/CerealsDB/search_reads.php), ensemble plants (http://plants.ensembl.org/common/Tools/Blast?db=core) and the International Wheat Genome Sequencing Consortium (IWGSC) sequence databases (http://wheat-urgi.versailles.inra.fr/Seq-Repository/BLAST) to obtain genome contigs for each EST. The IWGSC genome contig sequences were used to obtain CDS/transcript sequence using FGENESH annotation program. The transcript sequences were submitted to Pfam database for respective conserved domain corresponding to PHT1 (Pi:H+ symporter), PHT2 (Pi:H+ symporter), PHT3 (Pi:H+ symporter or Pi/OH- antiporter) and PHT4(Na+-dependent Pi cotransporter) family genes. For wheat PHTs identified with incomplete coding sequence, complete CDS and genomic structure were derived by genome assemblies using cereal DB, ensemble plants and IWGSC survey sequence databases and subsequent BLASTX analysis at NCBI. Information on chromosomal localization was obtained for all genes from IWGSC database. The naming of the wheat PHT genes were based on sequence similarity with rice orthologues.

The protein sequence alignment and construction of neighbour-joining phylogenetic tree was performed using MEGA 5^53^. Bootstrap values were calculated as a percentage of 1000 trials. The prediction of potential motifs in the wheat PHT family gene sequences was done using Multiple Em (Expectation maximization) for Motif Elicitation (MEME) program version 3.5.4^54^. Promoter sequences (1 kb upstream of start codon) for all *TaPHTs* were retrieved from IWGSC genomic contig sequences and screened for *cis*-elements using plant cis-acting regulatory DNA elements (PLACE) algorithm (http://www.dna.affrc.go.jp/PLACE/signalscan.html).

### Plant material

A bread wheat (*Triticum aestivum*) variety, C306 was grown in three replicates with adequate amount of nutrients at the research farm of National Agri-Food Biotechnology Institute (NABI). The main individual spikes of the biological replicates were tagged at the first days after anthesis (DAA). The tagged spikes were harvested at four main developmental stages i.e. 7, 14, 21, and 28 DAA and frozen in liquid nitrogen for RNA extraction. Seeds from transgenic wheat with lower phytic acid were used from the previous study^33^. To compare the expression of genes in aleurone (includes crease area), endosperm and embryo, these tissues were manually separated from 14 DAA seeds on dry ice and were frozen for further processing. For studying gene expression in different parts of wheat, tissues were collected from seeds, roots, shoots, leaves and flag leaf of wheat plants at the stage of 14 DAA.

For Pi starvation experiment, wheat plants with three biological replicates (each having 10–12 seedlings of 7 days old) for each mentioned conditions were grown using hydroponic culture carried out in growth chamber set at 20 ± 1 °C, 50–70% relative humidity and photon rate of 300 μmol quanta m^−2^ s^−1^ with 16 h day/8 h night cycle. Hydroponic system with Hoagland medium solution was used for phosphate starvation experiments. For high P and low P conditions, 1 mM KH_2_PO_4_and 10 μM KH_2_PO_4_ (additional 190 μM KCl) was used respectively. In each Phytabox^TM^ containing the growing seedlings nutrient medium was refreshed every day. For evaluating P uptake and gene expression, plants were grown in high and low P medium for 15 and 20 days, and subsequently roots and shoots were harvested for further analysis^16^.

### RNA isolation

Total RNA was isolated from 50–100 mg tissue from various tissue samples. RNA was isolated from the multiple stages of wheat seed development (7, 14, 21 and 28 DAA) and 14 DAA plant tissues (root, leaf, stem, flag leaf, rachis, glume, aleurone, endosperm and embryo) was done using the guanidine thiocyanate extraction method with Trizol^TM^ reagent (Invitrogen). For the seed tissue-specific study, 14 DAA seeds were subjected to manual dissection in the presence of dry ice with careful removal of embryo with a little cut on dorsal side of seed, followed by peeling off the aleurone and allowing embryo-less starchy endosperm to separate out. The collected aleurone tissue also contained crease region. As nucellar epidermis and integuments were too small, no clear observation was made concerning their fate. For whole-seed tissue collections, a minimum of fifteen seeds from three different plants were pooled together. The tissues were subsequently snap-frozen in liquid nitrogen. The trace amount of genomic DNA was eliminated by DNase I treatment using an RNase free kit (Ambion, USA).

### Quantitative real time PCR (qRT-PCR)

Two micrograms of DNA-free RNA was used for the first strand cDNA synthesis using the Transcriptor First Strand cDNA Synthesis Kit RT-PCR (Roche, USA) with random hexamer primers following the manufacturer’s guidelines. The qRT-PCR reactions were performed using gene-specific primers (Supplementary Table 4) by using QuantiTect SYBR Green RT-PCR Master mix (Qiagen) for 45 cycles on ABI 7700 Sequence Detector (Applied Biosystems, Foster City, CA, USA). The C_t_ values obtained were normalized against ELF-α, ARF and 18 S rRNAbecause their expression was shown to be consistent. At least two or three separate RNA preparations from the biological samples were used for the transcript expression analysis with four to five technical replicates for each cDNA sample. Target-specific product amplification was verified by melting curve analysis after every run. The relative transcript levels were determind by 2^−∆∆CT^ method^55^ for every cDNA samples.

### Assay of free phosphate and total phosphate in wheat seed tissues

Pi concentration in wheat seeds (7, 14, 21 and 28 DAA), 14 DAA seed tissues (aleurone, endosperm and embryo) and starvation samples (roots and shoots) was measured by molybdate-blue colorimetric method^56^. The 0.5 g of tissues in three biological replications was extracted in 0.5 ml of extraction buffer (10 mM Tris, 1 mM EDTA, 100 mM NaCl, 1 mM β-mercaptoethanol, and 1 mM phenylmethylsulfonyl fluoride, pH 8.0) as described by earlier^57^. Further, 0.7 ml of assay solution (Ascorbic acid, 10% and 0.42% ammonium molybdate in 1 N H_2_SO_4_) was added to 0.3 ml of sample solution (or to 0.3 ml of water for the blank) and incubated at 42 °C for 40 minutes. Pi concentrations were detected at the absorbance of 820 nm. For the standard preparations, 1 mM of KH_2_PO_4_ solution was used at different volumes (0, 5, 15, 20, 25, 30, 35, 40, 45 μL).

For total P analysis, approximately 100 mg powder was digested with nitric acid in the Microwave Reaction System (Mars 6, CEM Corporation, USA). P concentration was estimated in the digested samples using inductively coupled plasma mass spectrometry (ICP-MS; 77006AgilentTechnologies, Santa Clara, CA), following standard protocol.

## Additional Information

**How to cite this article**: Shukla, V. *et al*. Tissue specific transcript profiling of wheat phosphate transporter genes and its association with phosphate allocation in grains. *Sci. Rep.* **6**, 39293; doi: 10.1038/srep39293 (2016).

**Publisher's note:** Springer Nature remains neutral with regard to jurisdictional claims in published maps and institutional affiliations.

## Supplementary Material

Supplementary Tables and Figures
