# Supplementary material for: Tissue specific transcript profiling of wheat phosphate transporter genes and its association with phosphate allocation in grains
Source: Sci Rep. 2016 Dec 20;6:39293. doi: 10.1038/srep39293 (PMC5172359; doi:10.1038/srep39293)
Supplement: Supplementary Tables and Figures [file srep39293-s1.pdf]

Tissue specific transcript profiling of wheat phosphate transporter genes and its association with phosphate allocation in grains

Vishnu Shukla[1], Mandeep Kaur[1], Sipla Aggarwal[1], Kaushal Kumar Bhati[1,2], Jaspreet Kaur[3], Shrikant Mantri[1] and Ajay K Pandey[1]*

Author affiliations:

[1]National Agri-Food Biotechnology Institute (Department of Biotechnology), C-127, Industrial Area, S.A.S. Nagar, Phase 8, Mohali-160071, Punjab, India.

[2]Current Affliation: Copenhagen Plant Science Centre, PLEN, University of Copenhagen, Thorvaldsensvej 40, 1871 Frederiksberg C, Denmark

[3]Department of Biotechnology, University Institute of Engineering and Technology (UIET), Panjab University, Chandigarh, India

*Corresponding author: Dr. Ajay K Pandey, Scientist-D

Email: pandeyak@nabi.res.in ; pandeyak1974@gmail.com

Supplementary Table 1: Summary of 23 putative wheat phosphate transporters (*TaPHTs*), their chromosomal locations, uniprot ID's, domain information and previous names (past references). Sequences of underlined homeoloci (example, 3B for TaPHT4.5) were used for *in-silico* promoter analysis.

Name (Previous name)	Chromosome location	Length (amino acid)	Transmembrane domains	Uniprot ID	IWGSC ID	Reference
**CLUSTER I**						
TaPHT1.1 (TaPHT1.2)	4A, 4B, 4D	525	12	W5E578	Traes_4BL_8A75B60E2<br>Traes_4AL_959311BC9<br>Traes_4DL_229E76188	Davies et al., 2002,
TaPHT1.2	4A, 4B,4D	502	12	W5E3Z6	Traes_4BL_63BDFB322	Current study
TaPHT1.3	5A, 5D, 5B	497	12	W5FQX3	Traes_5DL_1B21D9A26<br>Traes_5BL_1CF10603C	Current study
TaPHT1.4	5B	566	12	W5F8C4	Traes_5BL_86CA8C1DD	Current study
TaPHT1.5 (TaPHT1.5)	5A, 5D, 5B	535	12	W5FKS5	Traes_5BL_FC28B66D8	Aziz et al., 2013
TaPHT1.6	5D	535	12	W5FS90	Traes_5DL_391DF2590<br>Traes_5DL_D473C031B	Current study
TaPHT1.7 (TaPHT1.8)	4A, 4B, 4D	536	12	A0A096UQG8	Traes_4AL_C56125840	Davies et al., 2002
TaPHT1.8	5D	536	12	W5FZ60	Traes_5DL_D473C031B<br>Traes_5DL_E15C27E9B	Current study
TaPHT1.9 (TaPHT1.1)	6A, 6B, 6D	533	12	W5GAF8	Traes_6AL_D2BE73957<br>Traes_6DL_057F2930B<br>IWGSC_CSS_6BL_scaff_196133:1-388	Aziz et al., 2013
TaPHT1.10	4A, 4B, 4D	470	8	W5E1D4	Traes_4BL_0F9E7D1F3	Current

						study
TaPHT1.11 (TaPHT1.11)	4A, 4B, 4D	553	12	W5ETT7	Traes_4DS_F2C36025E<br>Traes_4AL_D70AE8BE9<br>Traes_4BS_27217AD61	Aziz et al., 2013
TaPHT1.12	4A	528	10	W5DSM6	Traes_4AL_B174DBADB<br>Traes_4AL_072D29F34	Current study
TaPHT1.13	2A, 2B, 2D	530	12	W5BT34	Traes_2AS_A9B96E2B9<br>Traes_2AS_CE01C9B35	Current study
**CLUSTER II**						
TaPHT2.1 (TaPHT2.1)	6A, 6B, 6D	568	13	W5GWA8	Traes_6DL_544B74CBB1<br>Traes_6AL_10606E000<br>Traes_6BL_5C168B1DD	Guo et al., 2013
**CLUSTER III**						
TaPHT3.1	6A, 6B, 6D	305	5	W5G8R8	Traes_6AL_939CC781A<br>Traes_6DL_41A4CE766<br>Traes_6BL_CE9CC5475	Current study
TaPHT3.2 (TaPHT3.1)	4 A, 4B, 4D	378	5	W5EHP6	Traes_4AS_3CBD43E82<br>Traes_4DL_56122574F<br>Traes_4BL_32BED4269	Aziz et al., 2013
TaPHT3.3	2 A, 2B, 2D	380	5	W5BGN6	Traes_2BL_E80C4EE41<br>Traes_2AL_BBB5A17EE<br>Traes_2BL_E80C4EE41	Current study
**CLUSTER IV**						
TaPHT4.1	3A, 3B, 3D	467	12	A0A077RRV5	TRAES3BF034900010CFD_g<br>Traes_3DS_AC43888BB<br>Traes_3AS_4405E0087	Current study
TaPHT4.2	3A, 3B, 3D	498	12	W5DEB3	TRAES3BF142500030CFD_g<br>Traes_3AL_0A5D09D9D<br>Traes_3DL_DFACE051B	Current study
TaPHT4.3	1A, 1B, 1D	440	12	W5AHF8	Traes_1DL_A77D41F00<br>Traes_1AL_91970EBB9<br>Traes_1BL_401BAF780	Current study
TaPHT4.4	4A, 4B, 4D	574	12	W5DN82	Traes_4AL_3BE1CA5A8<br>Traes_4DS_12E3A8F5C<br>Traes_4AL_3BE1CA5A8	Current study
TaPHT4.5	3A, 3B, 3D	436	12	A0A077S0J7	TRAES3BF072600080CFD_g<br>Traes_3AL_2BD08DAA6<br>Traes_3DL_67B0AAC93	Current study

TaPHT4.6 (TaPHT4.1)	5A, 5B , 5D	540	12	W5FAE7	Traes_5BL_41F9FAA36<br>Traes_5DL_E85FB920C<br>Traes_5AL_8F5E57430	Aziz et al., 2013

Table S2: Analysis of promoter sequences of *TaPHT* genes (1kb sequences were retrieved from one homeoloci as mentioned in Table1)

cis – regulatory element	PHT 1.1	PHT 1.2	PHT 1.3	PHT 1.4	PHT 1.5	PHT 1.6	PHT 1.7	PHT 1.8	PHT 1.9	PHT 1.10	PHT 1.11	PHT 1.12	PHT 1.13	PHT 3.1	PHT 3.2	PHT 3.3	PHT 4.1	PHT 4.2	PHT 4.3	PHT 4.4	PHT 4.5	PHT 4.6
P1BS	2	2	0	0	2	4	0	0	2	0	4	0	0	4	2	3	0	2	0	0	0	0
CATATGGMSAUR	4	2	0	0	0	0	2	0	0	0	0	0	0	0	0	0	0	0	0	0	0	0
ACGTABREMOTIFA2OSEM	0	0	0	0	1	1	0	1	0	0	0	1	0	0	0	0	2	1	0	0	0	0
WBOXNTERF3	6	6	1	3	6	3	3	3	4	2	6	0	0	2	1	3	2	2	2	4	1	2
WBOXATNPR1	3	3	3	4	3	2	3	2	1	1	6	0	0	2	2	0	2	3	1	1	0	0
GARE1OSREP1	2	0	0	0	0	1	0	0	0	0	0	0	0	0	0	0	0	0	0	0	0	0
T/GBOXATPIN2	0	0	0	0	0	0	0	1	0	1	0	2	1	0	0	0	1	0	2	0	0	0
ACGTABOX	4	0	0	0	0	0	0	0	0	0	0	0	0	0	0	0	0	4	0	0	0	0
WBOXHVISO1	3	4	0	1	6	3	2	0	3	1	2	0	0	2	1	1	2	1	0	1	0	0
SURECOREATSULTR11	0	3	2	1	0	2	3	2	1	1	4	5	1	2	4	4	4	4	0	1	2	1
MYBCORE	6	6	4	6	6	9	6	4	6	6	7	3	2	5	5	5	1	3	1	3	6	4
ACGTATERD1	4	2	2	0	4	4	8	4	2	4	0	6	2	2	10	4	6	13	4	0	0	2
CURECORECR	12	8	2	2	2	6	6	4	12	2	6	4	2	4	10	6	0	6	2	6	4	6
MYCCONSENSUSAT	10	6	8	8	14	10	12	2	18	8	12	16	6	12	8	8	8	18	8	10	14	8

Legends:

Pi-responsive **Hormone-responsive** **Sugar-responsive** **Stress - responsive**

Supplementary Table 3: Identifiers (Genbank, swiss-prot and Locus IDs) of *Arabidopsis* and Rice that were used in the current study to identify wheat phosphate transporters.

Transporter Name	Amino acid residue	Accession numbers
OsPT1	527	AK071903
OsPT2	528	AK108907
OsPT3	526	AK071903
OsPT4	538	AK101380
OsPT5	548	AK101380
OsPT6	534	AK119787
OsPT7	526	AK121484
OsPT8	541	AK101170
OsPT9	211	AK109109
OsPT10	552	AK109109
OsPT11	555	AK062362
OsPT12	541	AK106694
OsPT13	508	AK101380
OsPT14	572	AK067498.1
OsPT15	368	AK069611
OsPT16	374	AK058971
OsPT17	360	AK069397.1
OsPT18	368	AK066979.1
OsPT19	278	Os09g28160
OsPT20	323	AK241216
OsPT21	529	AK067683.1
OsPT22	519	AK063954.1
OsPT23	535	AK120548.1
OsPT24	591	AK065287.1
OsPT25	428	AK121325.1
OsPT26	439	AK071259.1
AtPHT1.1	524	Q8VYM2
AtPHT1.2	524	Q96243
AtPHT1.3	521	O48639
AtPHT1.4	534	Q96303
AtPHT1.5	542	Q8GYF4
AtPHT1.6	516	Q9ZWT3
AtPHT1.7	526	Q494P0
AtPHT1.8	541	Q9SYQ1
AtPHT1.9	532	Q9S735
AtPHT2.1	587	Q38954
AtPHT3.1	375	NM_121407.2
AtPHT3.2	363	NM_114744.3
AtPHT3.3	309	NM_127282.2
AtPHT4.1	529	NM_128519.5
AtPHT4.2	535	NM_129362.3
AtPHT4.3	519	NM_114565.2
AtPHT4.4	541	NM_116261.4
AtPHT4.5	471	NM_122045.3
AtPHT4.6	428	NM_123804.3

Supplementary Table 4: Primers used in the current study

Gene name	Homeoloci amplified	Forward primer (5' to 3')	Reverse Primer (3' to 5')	amplicon (base-pairs)
TaPHT1.1	A,B,D	CTGCACCGCCATTCTACATCGACG	CTTGTGGTCGCTAGCAAGTGCACC	316
TaPHT1.2	A,B[#]	GAGGCCGACTACGTGTGGCG	GCACGCTCACCCTGCACATCCT	197
TaPHT1.3	A,B,D	CTGCGCCGGCACGGGC	CGTGAACCAGTAGCCCGGCAC	219
TaPHT1.4	B*	ATCCTCATGCTCGGCGCCCTC	TCCGCCGCGGCTCACGAG	189
TaPHT1.5	A,B,D	GCCGACGACCGCAACTCGTTC	CGTGAACCAGTAGCCCGGCAC	261
TaPHT1.6	D*	GTCCACCCCCGGCTCCGTC	GCGACGCGGTCGTCCTGGTA	255
TaPHT1.7	A,B,D	GACAAGATGGGACGCAAGAGCGTC	CGAAGACAGCGGCGATGAAGGC	232
TaPHT1.8	D*	TCGCGCACACCCCCAAGAGC	GCGACGCGGTCGTCCTGGTA	259
TaPHT1.9	A,B,D	GCGTCCTGGTTCGTGCTCGACA	TGCGTCAGTTGGTGGTAGAAGCCG	290
TaPHT1.10	A,B[#]	GCAGGCCGACTACATGCCGGA	AGGAACCACGCCGTCGCCGT	225
TaPHT1.11	A,B,D	CTGGCTCGGCTTCGGCATCG	GCATCCCACGCCGGTGCAG	198
TaPHT1.12	A*	TGCGGCGCTCACGTACTACTGG	AGGAACCACGCCGTCGCCGT	243
TaPHT1.13	A,D[#]	CAAGACGCAGTGGTACCACTTCACG	ACCGCTTCTTCCGGCCCATCTTG	257
TaPHT2.1	-	-	-	Not studied
TaPHT3.1	A,B,D	GCCTGGATTTGCTCGTGGGTTGAG	CAATGTAGCCACCAGCAAAGCTGATAC	248
TaPHT3.2	A,B,D	TAGCAAGTACAGGGACATCTCGTCTG	TCTGCAATTACCTCAGCCGACGCC	240
TaPHT3.3	A,B,D	GATTGATCCAGCCAAGTACAAGAGCATC	GGCAGAGGGCAACATCTGCAATAACCT	263
TaPHT4.1	A,B,D	CTCTGCAACATGGACCGCGTAAACAT	GCGTAAGAGCTGTGGCGACCGA	217
TaPHT4.2	A,B,D	TCTGTGGGTGCTGGTGTGGATATCT	CCAGGTCGGCCATTTAGAAAGTAGG	181
TaPHT4.3	A,B,D	GCTGGACGAAGTAAATCAAAAGTCAAAGG	GGACTCGCACAATAGATAAGCCGGATT	289
TaPHT4.4	A,B,D	AAGCGCATGACCATCGTCGTCCTC	TGCCTCCAAGAATCTGAGTGAGAAGGTA	181
TaPHT4.5	A,B,D	GTGTAGCACAAGGTTTCATATTTCCTGC	CAACAACCCCTACTGATTGCGCCTTAAT	224
TaPHT4.6	A,B,D	TACAAGAACTACAGCCAGGTGACAATG	GAATGTTGGAAGCCAAGACAAGCAAG	251
TaPHR1	A,B,D	TTCCCAGGTTCGGCTGATG	CTGCCCACCATTCATTTTGTT	127
TaSPX1	A,B,D	GACGCTGTGACACACGATAC	GAAACAGGTGAGGTCCTGGT	240
TaPHO2	A,B,D	TCTCTGGTGACTGCGTAGTTATGGG	GGAGGGATGAACAATCCTCACGCG	180
TaIPS1	A,B,D	TGGTGCGAGTCTTCAGTGTCGTGTAA	CAAGCAGGATCATACACACACACCCAT	200
TaPHO85	A,B,D	CCGGTCGGCCTCTCTTCCCGGG	GTTCAACAAGTCCAGACCGAGAGG	202

* - Chromosomal location was confirmed using respective null line with homeolog sequence-specific primers; # - Contig sequence was not present in IWGSC database

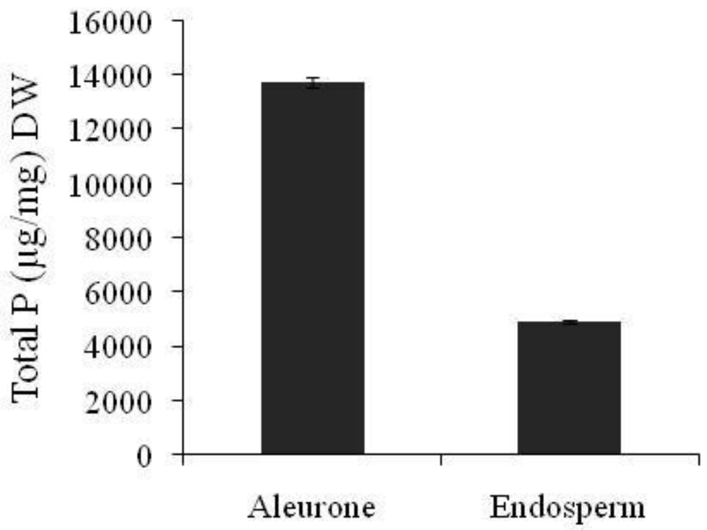

Supplementary Figure 1: Total phosphorous (P) content in 14 DAA Aleurone (Al) and Endosperm (En) estimated using ICP-MS.

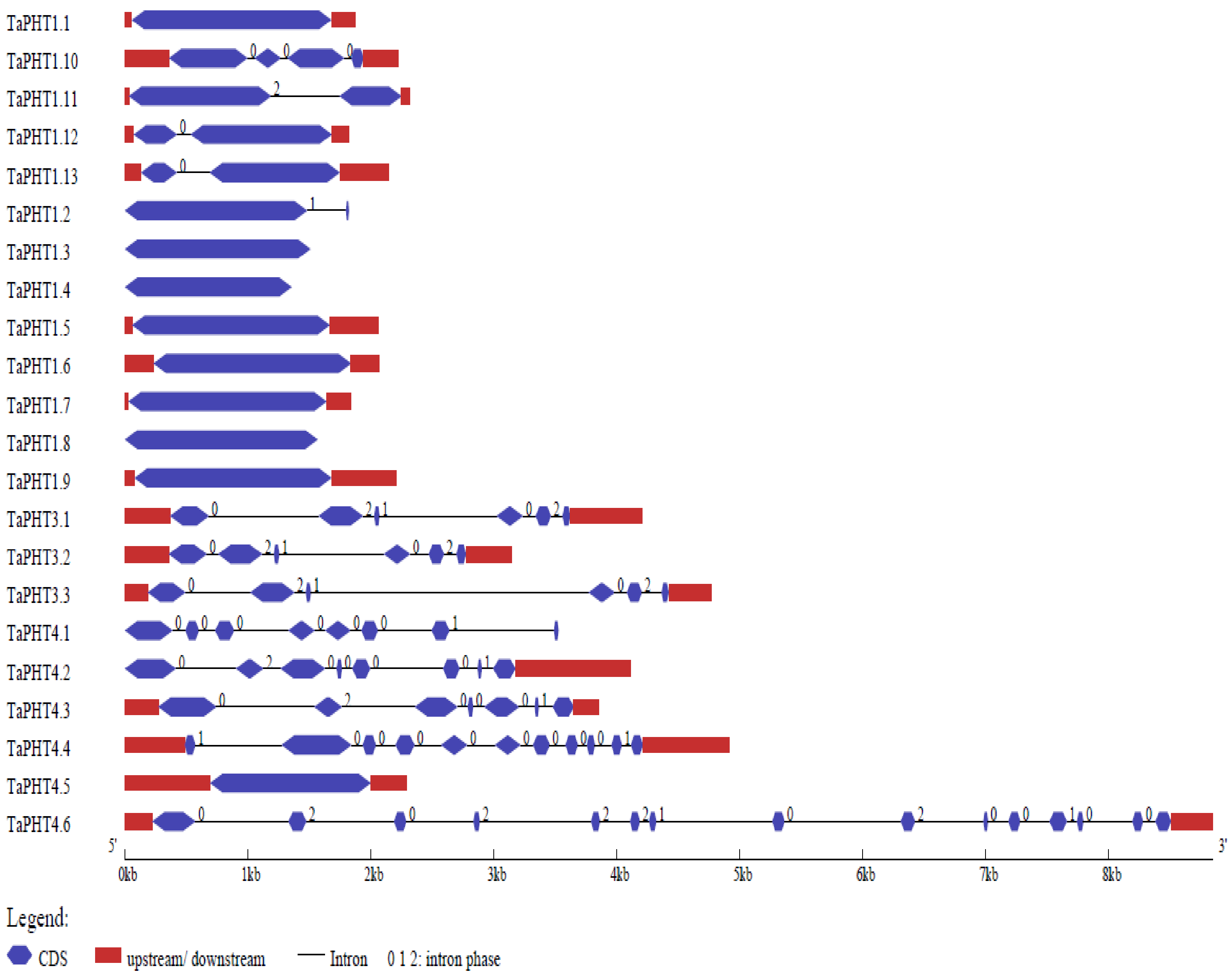

Supplementary Figure 2: Genomic structure of *TaPHTs* showing 5' & 3' UTR, Intron/exon arrangements with phases and CDS region. Sequences were analyzed online using Gene structure display server (GSDS 2.0).

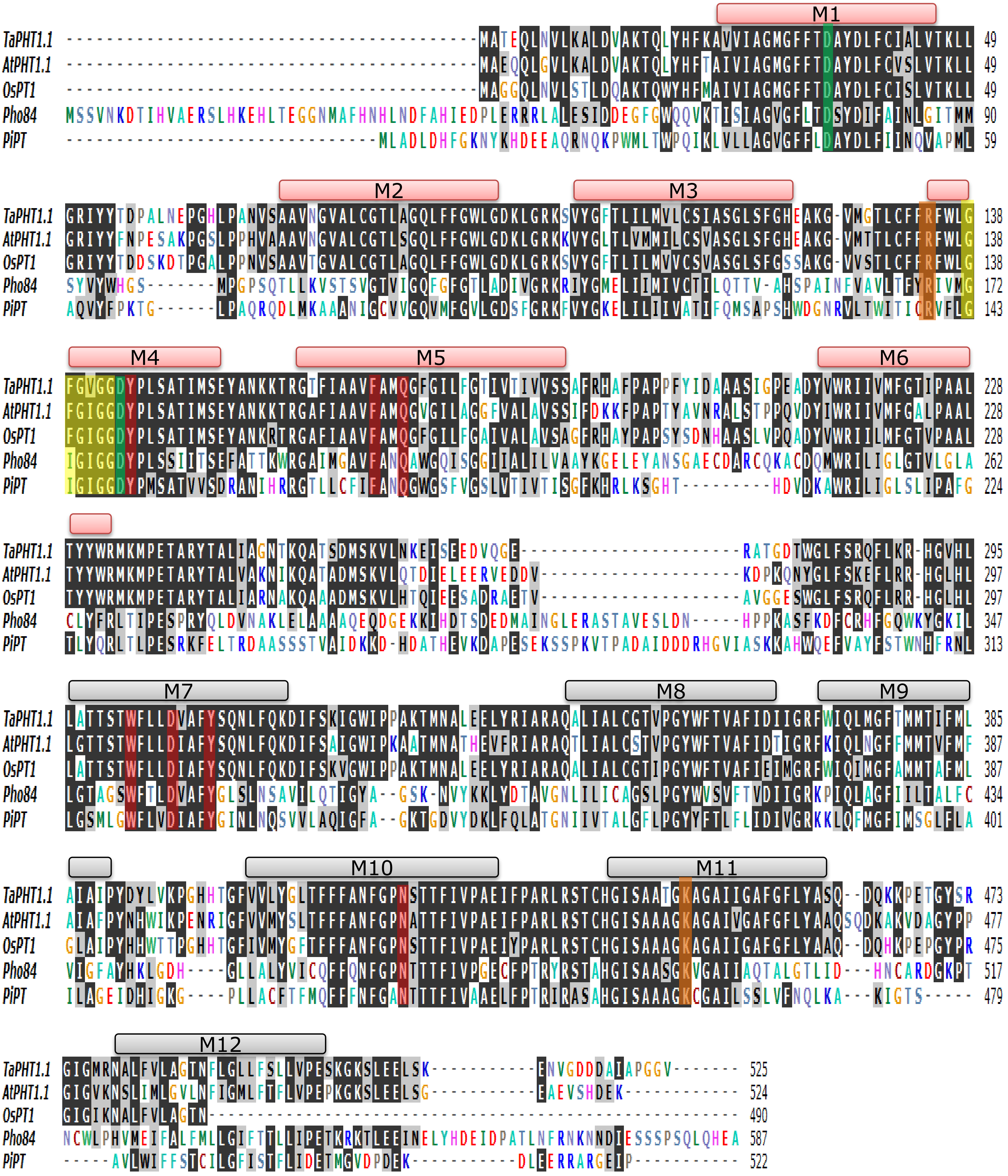

Supplementary Figure 3: Alignment between sequences of hight affinity phosphate transporters belonging to PHT1 family from *Piriformis indica*, *Saccharomyces cerevesiae*, *Oryzae sativa*, *Arabidopsis* and *Triticum aestivum*. The alignment was constructed using PROMALS3D and further analyzed with Phobius. Red and Grey tubes represent alpha-helices in N-domain and C-domain respectively *w.r.t TaPHT1.1* protein sequence. Conserved residues are highlighted with colors such as, phosphate binding domain shaded with pale red, glycine-rich motifs shaded with pale yellow, negatively charged residues shaded with pale green and other important residues are in pale brown. Information of conserved residues was collected from the work done by Pedersen *et al.*, 2013.

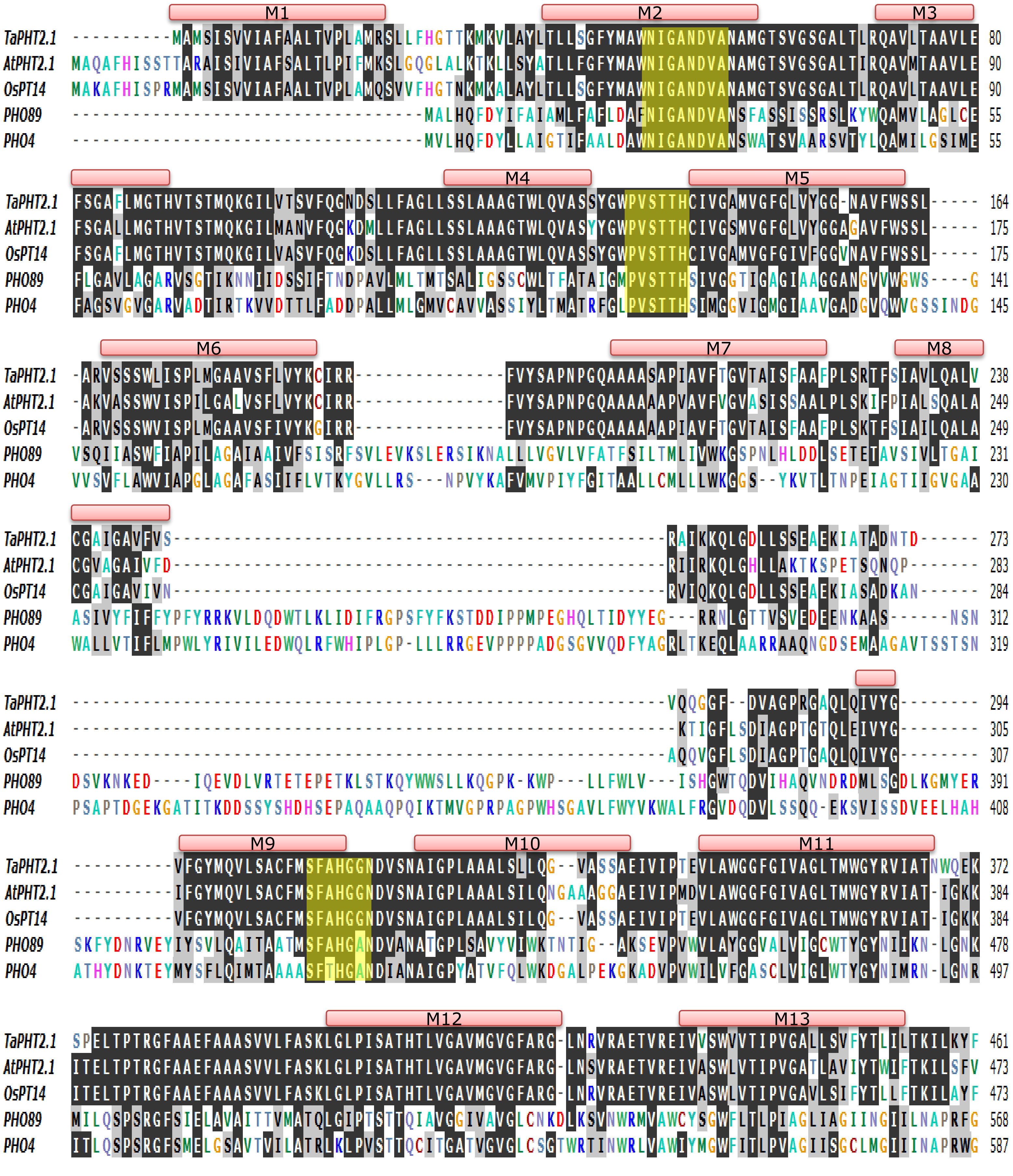

Supplementary Figure 4: Protein sequence alignment between plant PHT2.1 transporters with sodium-dependent Pi transporter Pho89 from *Saccharomyces cerevesiae* and PHO4 from *Neurospora crassa*. The alignment was constructed using PROMALS3D and further analyzed with Phobius and Transporter classification database. Red tubes represent transmembrane helices w.r.t *TaPHT2.1* protein sequence. As crystal structure of PHT2 sub-family member is not available domain responsible for Pi binding are not highlighted. Although, conserved residues are highlighted with pale yellow.

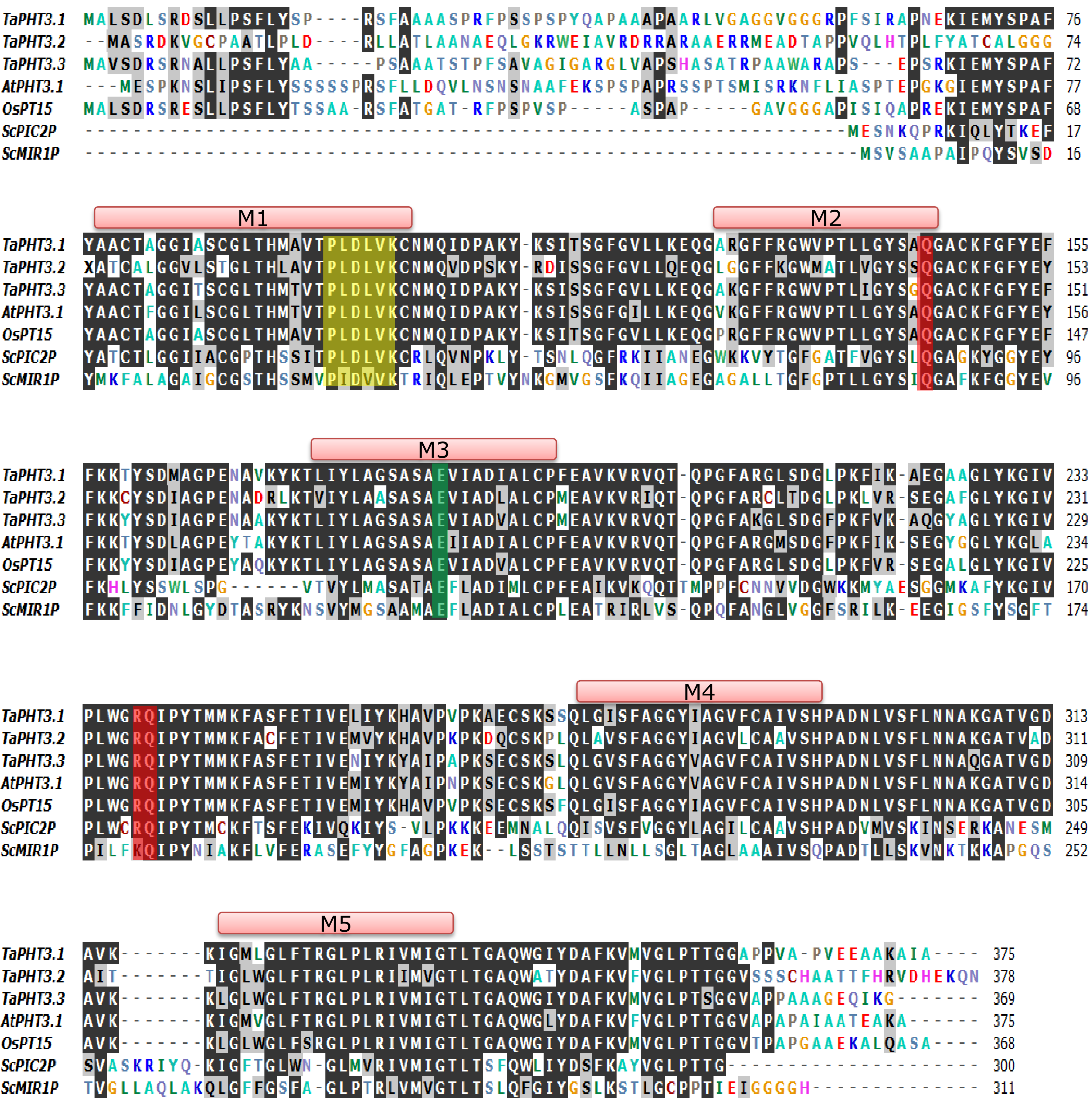

Supplementary Figure 5: Protein sequence alignment between plant PHT3 sub-family transporters (*Oryzae sativa, Arabidopsis* and *Triticum aestivum)* with mitochondrial carrier family proteins such as *ScPIC2P* and *ScMIR1P* from *Saccharomyces cerevesiae*. The alignment was constructed using PROMALS3D and further analyzed with Phobius. Red tubes represent transmembrane helices *w.r.t TaPHT3.1* protein sequence. Functionally important conserved residues are highlighted with colors such as, phosphate binding site shaded with pale red, signature residues involved in forming salt-bridges shaded with pale yellow, residue involved in proton coupling shaded with pale green.

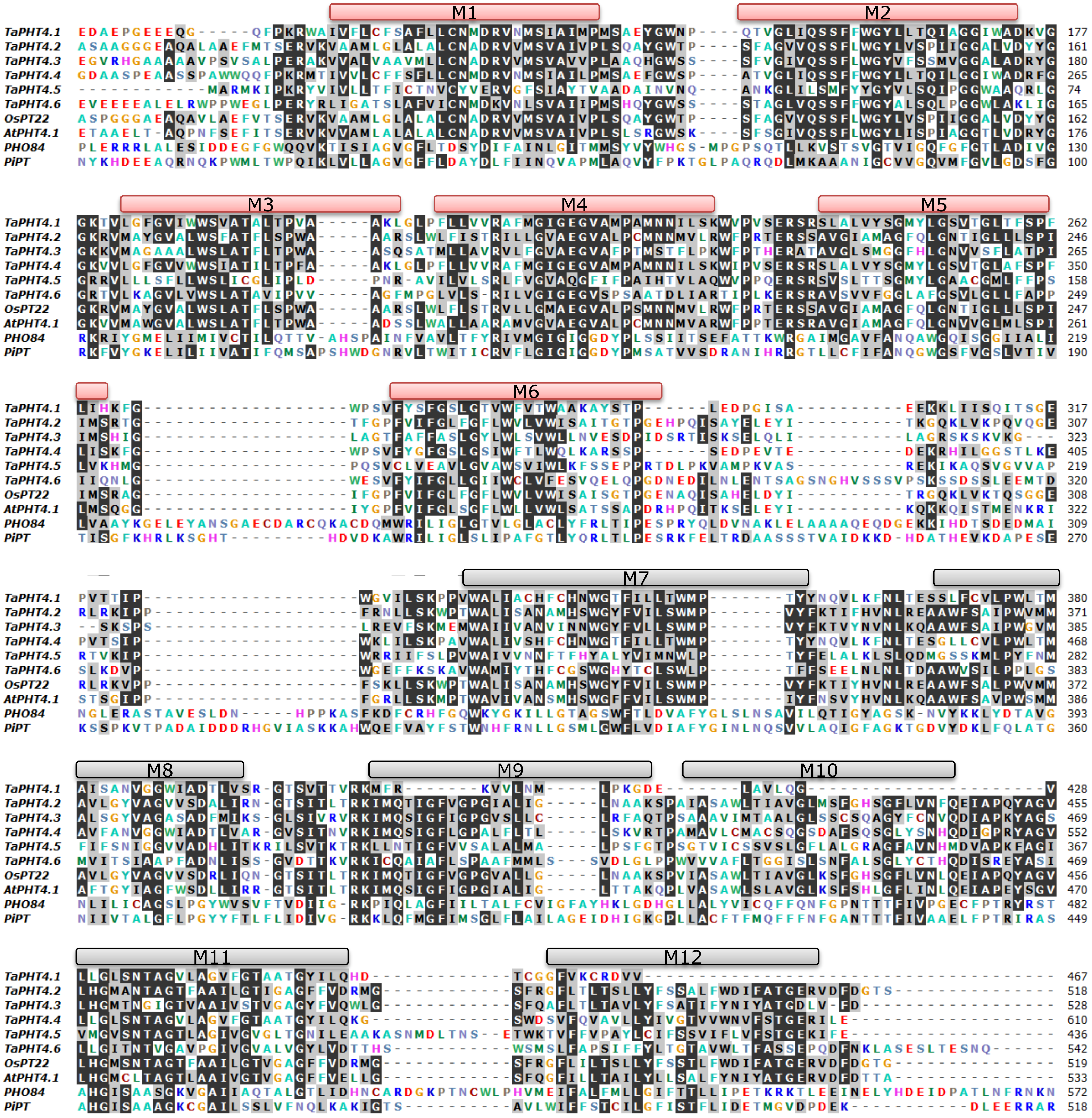

Supplementary Figure 6: Protein sequence alignment between plant PHT4 sub-family transporters with high affinity transporters such as *Pho84* and *PiPT* from *Piriformis indica* and *Saccharomyces cerevesiae*. For Wheat all members of PHT4 members were included (*TaPHT4.1 – TaPHT4.6*). The alignment was constructed using PROMALS3D and further analyzed with Phobius and Transporter classification database. Red and Grey tubes represent transmembrane helices in N-domain and C-domain respectively *w.r.t TaPHT4.1* protein sequence. Functionally important conserved residues that are present in *PiPT* (Pedersen et al., 2013), were not conserved in PHT4 sub-family members. This reflects the affinity of plant PHT4 members towards Pi uptake through membrane. Although somewhat conserved residues were highlighted with pale red.

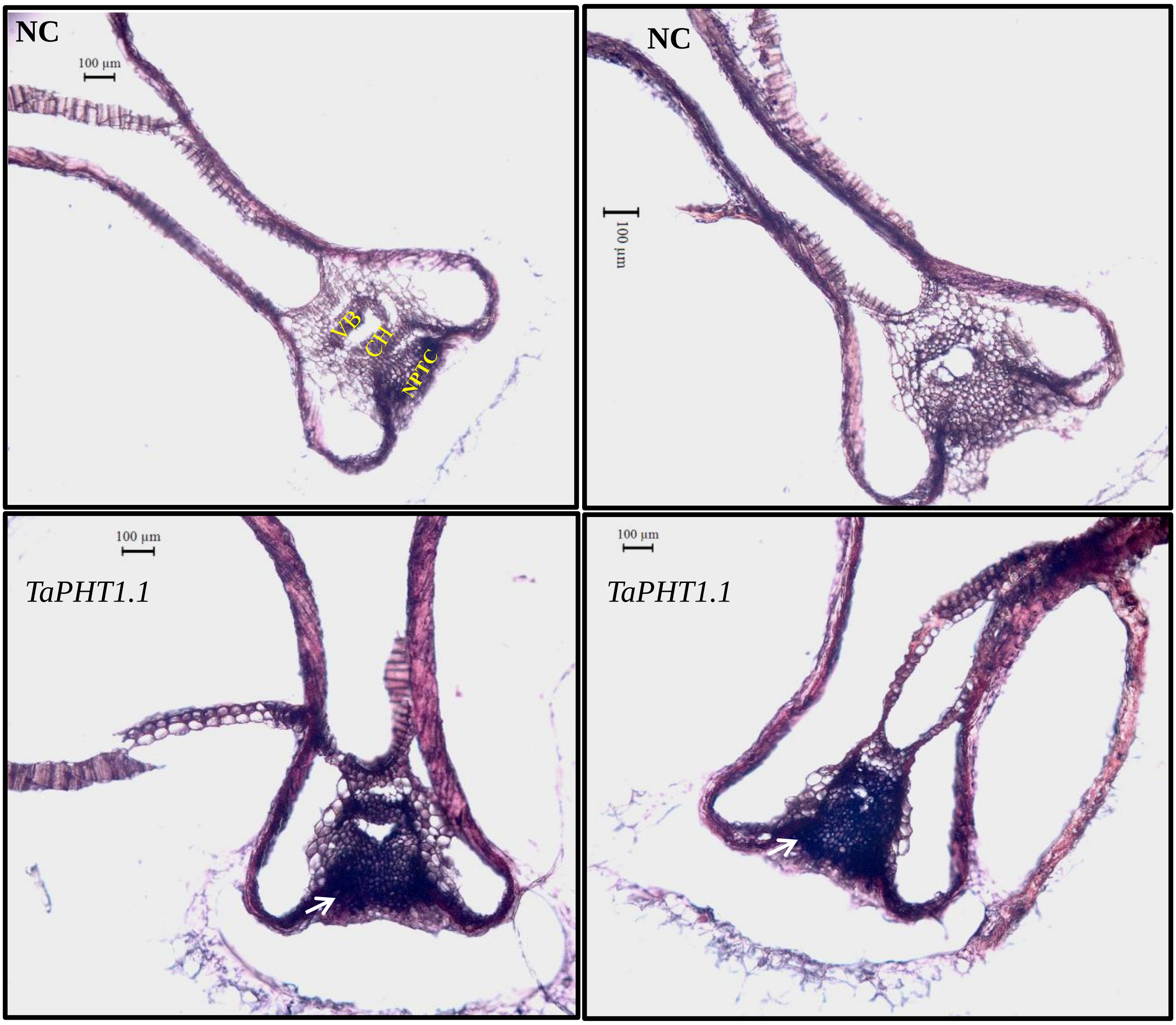

Supplementary Figure 7: *In-situ* PCR for *TaPHT1.1* in section of wheat grains (14 DAA) for expression analysis. In-situ PCR was performed with labeled dUTPs and tissue was subjected for cDNA and PCR analysis. Blue staining demonstrates presence of cDNA (lower panels) while a brown colour indicates absence of amplified target cDNA within tissues (upper panels). Expression data indicates high expression of *TaPHT1.1* in the NPTC (nucellar projection transfer cells) and chalazal (CH) region of the crease area. NC- negative control, VB-vascular bundle.
